# Overcoming a false-positive mechanism in RapidFire MRM-based high throughput screening

**DOI:** 10.1016/j.slasd.2025.100252

**Published:** 2025-09

**Authors:** De Lin, Lesley-Anne Pearson, Shamshad Ahmad, Sandra O’Neill, John Post, Colin Robinson, Duncan E. Scott, Ian H. Gilbert

**Affiliations:** Drug Discovery Unit, School of Life Sciences, University of Dundee, Dundee, DD1 5EH, UK

## Abstract

False-positives plague high-throughput screening in general and are costly as they consume resource and time to resolve. Methods that can rapidly identify such compounds at the initial screen are therefore of great value. Advances in mass spectrometry have led to the ability to screen inhibitors in drug discovery applications by direct detection of an enzyme reaction product. The technique is free from some of the artefacts that trouble classical assays such as fluorescence interference. Its direct nature negates the need for coupling enzymes and hence is simpler with fewer opportunities for artefacts. Despite its myriad advantages, we report here a mechanism for false-positive hits which has not been reported in the literature. Further we have developed a pipeline for detecting these false-positive hits and suggest a method to mitigate against them.

## Graphical abstract

**Figure:**
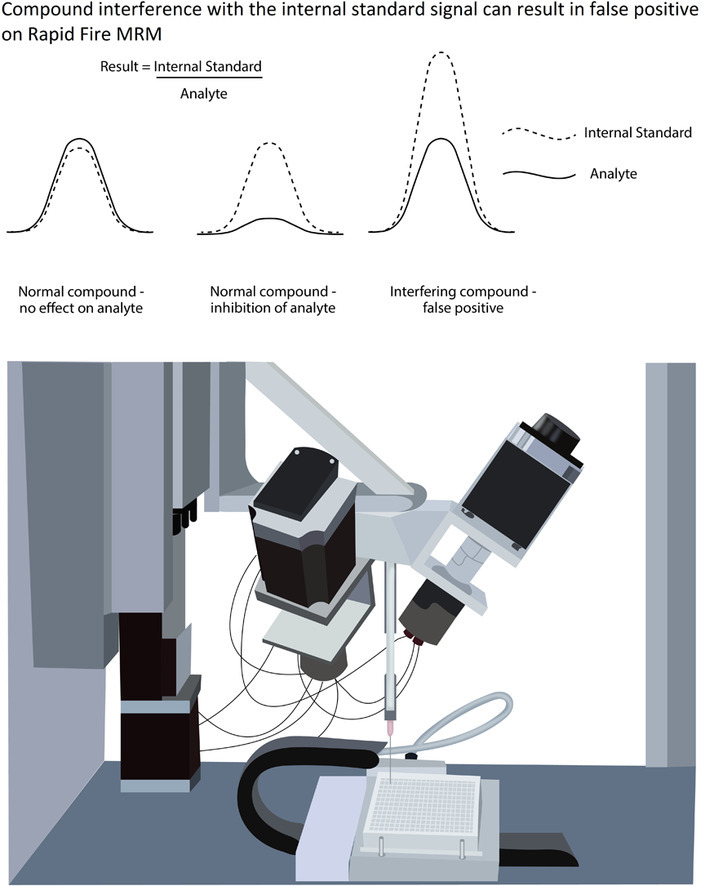

## 1 Introduction

RapidFire mass spectrometry (RF-MS) is a high-throughput mass spectrometry technique that allows for the analysis of large numbers of samples in a short amount of time. This has made it a valuable tool for high-throughput screening (HTS) applications in various fields, such as drug discovery, metabolomics, and proteomics and is compatible with various sample matrices (cell lysate, plasma, tissue etc.) [[1], [2], [3], [4]]. RapidFire coupled with triple quadrupole (QQQ) mass spectrometry for targeted multiple reaction monitoring (RF-MRM) has been used in target-based screening for screen large libraries of compounds in various targets including *Trypanosoma brucei* AdoMetDC [5], HIV-1 Protease [6] and WIP1 Phosphatase [7]. The ability to assay the activity of a target by direct detection of increase of product, or depletion of substrate is a technique that is gaining in popularity, with the expectation that the removal of complex coupling reactions reduces the risk of assay interference. For example, in a previous work (Pearson et al. 2025) we measured the activity of the SAM (S-adenosylmethionine) dependent methyltransferase RNMT [8] using direct detection of the product SAH (S-adenosylhomocysteine) by RF-MRM, after having found the commercially available ‘coupled reaction’ was prone to false positives.

An internal standard is typically added to a sample prior to mass spectrometry analysis to correct for variations in the sample preparation, instrumental parameters, and matrix effects that can affect the response of the analyte [9]. This is particularly useful in RF-MRM analysis where the sample matrices can be complex and variable, leading to variations in the analyte response [10]. The signal ratio of the analyte to the internal standard is reported as a ‘normalised response’ for quantification. The use of internal standards can also improve the precision, accuracy, and sensitivity of RF-MRM analysis. The internal standard is chosen such that it has same or similar chemical and physical properties as the analyte but can be distinguished from the analyte by its mass-to-charge ratio (*m/z*) in the mass spectrometer. A stable isotope labelled (SIL) analogue is believed to be most appropriate internal standard in a quantitative mass spectrometry [11]. However, the signal of the internal standard is potentially affected by compounds with the same molecular weight, especially in large library compound screening for drug discovery (where Lipinski’s ‘Rule of 5′ for ‘drug like’ molecules would suggest a molecular weight below 500 Da [12]) and when using low resolution mass spectrometry instruments (e.g. quadrupole). This likelihood may increase when the analyte being detected is of a similar size, for example in SAH in methyltransferases [13] or ADP (Adenosine diphosphate) in kinases [14], and the selection of the internal standard is matched to it (as we have described above).

We previously published a method for screening compounds in a relatively high-throughput mode against the SARS-CoV-2 protein nsp14 using RF-MRM [11] (Pearson et al. 2021), which directly detects the SAH product as described above. We have now used this to assess the potency of a range of drug-like molecules. In addition, we used a luminescence-based MTase-Glo assay (Promega) as an orthogonal assay to confirm the activity of hits identified in the RF-MRM assay. This detects the presence of SAH with a coupled reaction to convert the SAH to ADP, and then a luciferase to produce light (see figure S6 for a workflow diagram comparing the two processes). In the process of testing compounds in the RF-MRM assay we identified a number of false positives with a previously undescribed mode of interference with the RF-MRM. In this paper we elucidate this mode of interference as well as offering possible ways to mitigate it.

## 2 Materials and methods

All aqueous solutions were prepared with deionized water (Millipore, Watford, Hertfordshire, UK). All reagents were purchased from Sigma Aldrich (Gillingham, Dorset, UK) unless otherwise stated. Full-length SARS-CoV-2 nsp14 protein (DU66418) was supplied by the MRC-PPU (Dundee, UK). Nsp14 was cloned in fusion with a cleavable N-terminal GST fusion in a pGEX6P1 vector and expressed in *Escherichia coli*. Nsp14 was purified by batch purification using GSH-Sepharose beads and the tag was cleaved by PreScission protease. Cleaved nsp14 at 1.23 mg/mL was delivered in 50 mM Tris, pH 7.5, 150 mM NaCl, 270 mM sucrose, 0.1 mM EGTA, 0.03 % Brij-35, 0.1 % β-mercaptoethanol. Peptide IAYLKK*AT was synthesised at Cambridge Peptides by solid-phase synthesis. K* refers to ^13^C and ^15^N-enriched lysine (labelled on all carbons and nitrogens).

### 2.1 RapidFire assay

We have previously reported the use of RF-MRM to assess the potency of compounds against the methyltransferase activity of nsp14 from SARS-CoV-2 [15]. The protein catalyses the methylation of the N7-guanosine of RNA using S-adenosylmethionine (SAM), which is converted to S-adenosylhomocysteine (SAH). Briefly, enzyme and substrates (SAM (*S*-(5′-adenosyl)-l-methionine chloride hydrochloride [Cayman Chemical, Ann Arbor, MI]) and cap (G(5′)ppp(5′)G sodium salt [New England Biolabs, Ipswich, MA])) were incubated together along with the compound in 384 well clear, flat bottomed plates to a final assay volume of 20 µL prior to quenching the reaction with 1 % formic acid containing an internal standard of d4-SAH at 0.03 µg/mL. Compounds were tested in singlicate for a 10- point dose response curve [15]. The injection volume was 33 µL with 11 s (0.18 mins) per sample. Results were expressed as a normalised response: a ratio of the integrated peak (obtained from the RapidFire Integrator software) of the detected analyte, SAH, to that of the internal standard, d4-SAH.

This procedure was briefly modified later to allow comparison of the effect of a different internal standard by adding the peptide IAYLKK*AT as well as the original d4-SAH in the quenching solution. Results from these experiments were expressed as one of two possible normalised responses: a ratio of the detected analyte, SAH, to the original internal standard, d4-SAH, or the test internal standard, IAYLKK*AT. For both of these, percent effect was calculated by comparing the compound well results to the 100 % effect or 0 % effect of the enzyme in the control wells.

### 2.2 MTase-Glo assay

The MTase-Glo assay (Promega Corporation, Madison, WI, Cat. number V7601) was used as an orthogonal method of measuring nsp14 activity for confirming hits from the screen. Buffer conditions, proteins and substrate concentrations were kept the same as in the previously reported Rapid Fire assay [15]. Compounds were tested in singlicate for a 10- point dose response curve. The assay was carried out in white flat bottom assay plates and was stopped by the addition of MTase-Glo reagent (1x) at the end of the incubation time. Plates were then incubated in the dark for 30 min prior to the addition of the MTase-Glo Detection Reagent. The signal was allowed to develop for 30 min in the dark and plates were then read using a PHERAstar FS instrument to detect luminescence.

### 2.3 Pipeline pilot analysis

An automated script using BIOVIA Pipeline Pilot (BIOVIA Pipeline Pilot, ver. 20.1.0.2208, San Diego: Dassault Systèmes) and accessed via the webport was used to convert the internal standard and analyte data to the normalised response, as well as outputting the data in a standard 384 plate format. This normalisation serves to confirm successful sipping, successful detection of analyte by the RapidFire and smooth any well-to-well variability.

Following the identification of the false positives as described in this paper, a further script was written which automatically flags for further review any compound wells where the value for the internal standard is >3 standard deviations over the median for that plate. For details of these scripts, please contact the authors.

## 3 Results and discussion

**Fig. 1:**
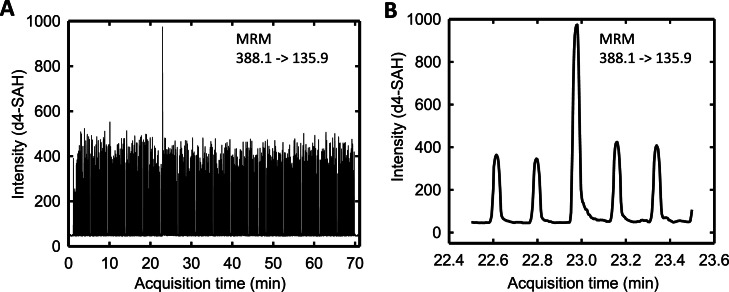
Example trace of high internal standard signal intensity. (A) Multiple reaction monitoring (MRM) chromatogram of d4-SAH from a 384-well nsp14 assay. (B) Zoomed-in view of the MRM chromatogram between 22.5 and 23.5 min. The injection volume was 33 µL, with a run time of 11 s (0.18 min) per sample.

**Fig. 2:**
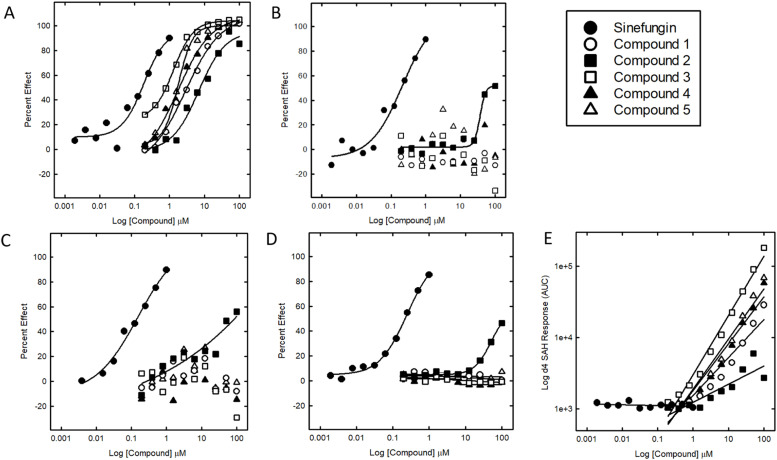
Dose response curves of the 5 test compounds and the Sinefungin control tested in singlicate. All percent effects (PE) are calculated with reference to 100 % effect (0 nM nsp14+DMSO) and 0 % effect wells (5 nM nsp14+DMSO). A. PE using SAH/d4-SAH normalised signal. B. PE using SAH signal (ignoring d4-SAH internal standard). C. PE using SAH/peptide IAYLKK*AT normalised signal. D. PE in MTase-Glo assay. E. d4-SAH signal.

With the reported RF-MRM nsp14 assay [15] in hand, we have screened libraries of compounds in a high-throughput manner for nsp14 inhibitors. In line with common HTS practices, we utilised the high-throughput capabilities of the RF to enable us to test large numbers of compounds at a single concentration to quickly identify potential hits, or chemical starting points, which were then evaluated with further replicates and progressed through the screening pipeline. Typically, the deuterated product d4-SAH is used as an internal standard in the experiment as it possesses similar properties in the mass spectrometer to that of the analyte product SAH. The expected behaviour is that the SAH signal will decrease with increasing inhibitor concentration, with the internal standard d4-SAH remaining at a constant level. The SAH signal is conveniently normalised to the internal standard d4-SAH signal as a ratio, to gauge the degree of nsp14 inhibition. During the process of validating hits from this nsp14 screen, we observed a small set of compounds showing aberrantly high values for the internal standard d4-SAH when viewing live data collection via the Agilent MassHunter Workstation Data Acquisition Software (Fig. 1). An automated analysis of these compounds showed well-behaved sigmoidal curves (Fig. 2A). However, when these compounds were subsequently tested in an orthogonal activity assay (MTase-Glo) they were found to be inactive (pIC50 <4)or had a potency reduced by ten-fold (pIC50 of 4.1 from a previously tested result of 5.1) (Fig. 2D).

To further understand the mechanism of these RF false-positive compounds, we re-examined the associated RF data in more detail. Inspection of the unconverted RF data revealed that the false-positive samples showed an unexpected dose-dependent increase in detected d4-SAH, rather than constant, and no dose-dependent decrease in the signal due to SAH, or a reduced decrease. When the SAH signal is normalised to the increasing d4-SAH signal, this gave the appearance of inhibition due to the compound-dependent decreasing SAH:d4-SAH ratio. If the analysis ignores the internal standard, and instead the percent effect is calculated using the effect on the SAH signal alone, these compounds showed either no apparent, or reduced inhibition, with a maximum percent effect not exceeding 55 % (see Fig. 2B), meaning that a compound previously designated as active (pIC50>4, max PE>50) is now defined as either inactive or weakly active. Examples of compound structures which displayed this interference and their precursor and predicted product ion *m/z* values are shown in Table 1.

**Table 1:** Compound structures and corresponding theoretical precursor ion with isotopic distribution and related product ion found in the d4-SAH measurement condition at precursor *m/z* 389.2 (**N-S5**). Fold increase in d4-SAH is calculated using the mean of the d4-SAH in the control wells vs the detected d4-SAH in the top concentration of the compound wells. Dashed lines on the structures indicate potential cleavage sites leading to product ion formation.

Compound	Structure	precursor exact mass	Theoretical Precursor ion m/z (%) (M/M+1/M+2)	Product ion (m/z)	Fold increase in ‘d4-SAH’
**d4-SAH**		388.15	**389.2 (100)**/390.2 (15) /391.2 (5)	135.9	n/a
**1**		387.02	388.1 (100)/**389.1 (20)**/390.1 (98)	136.2	27.8
**2**		386.05	387.1 (100)/388.1 (22)/**389.1 (37)**	136.1	2.5
**3**		388.08	**389.1 (100)**/390.1 (19)/391.1 (6)	136.2	178.9
**4**		386.12	387.1(100)/388.1(24)/**389.1(10)**	136.1	57.3
**5**		388.08	**389.1(100)**/390.1(20)/391.1(37)	136.0	70.2

Strikingly, all the false-positive hits have not only a precursor ion very close in mass to the internal standard d4-SAH but also a product ion that also closely matches the product ion of d4-SAH. Retesting of these compounds with an alternative internal standard (peptide IAYLKK*AT) showed that the false-positive compounds were indeed either completely inactive or had reduced (from pCI50 of 5.1 to a pIC50 of 4.1) activity as expected. It seems therefore that the observed original levels of inhibition with d4-SAH as an internal standard for these molecules was an artefact, caused by interference of the test compound and the detection of d4-SAH (see Fig. 2). It is worth noting that compound 2 displayed some inhibition against nsp14 once the interference mechanism was eliminated. Although this compound did display a dose dependent increase in apparent d4-SAH detected by the RapidFire (Fig. 2E), this was lower than that of the other compounds, and it still shows detectable inhibition when tested using the alternative internal standard (Fig. 2C) and the MTase-Glo orthogonal assay (Fig. 2D). This is another example of the impact that certain types of interference can have as, although not completely a false positive, the true activity of the molecule is masked by its interference. This could potentially impact the design of more potent analogues and understanding of its SAR (structure activity relationship), as well as introducing the risk that any new molecules from this starting point would only be optimising towards its interference.

When screening small molecule libraries using RF-MRM, it may not necessarily be easy to predict if a compound is likely to interfere in this way based upon the compounds structure. However, if the internal standard used is close in molecular weight to that of the test compound, then it is advisable to consider this interference mechanism and treat any observed inhibition with caution. An orthogonal assay or rescreening the hits with a different internal standard will help to distinguish true versus false positives. Alternative mass spectrometry techniques can be employed to address these challenges as well. Time-of-flight (ToF) or orbitrap analyzers providing high mass resolution and accuracy enable more confident differentiation between closely spaced *m/z* signals. Ion mobility spectrometry (IMS), particularly when coupled with QQQ or ToF instruments, can separate ions based on their shape and charge in the gas phase [16], offering an extra dimension of separation that helps distinguish isobaric or isomeric species. Additionally, Matrix-assisted laser desorption/ionization (MALDI) offers high tolerance to complex mixtures and spatial separation in solid-phase formats, reducing overlap in signal [17,18]. Together, these approaches can greatly enhance selectivity and reliability in assays where internal standard interference is a concern.

Although our initial identification was revealed by visual inspection of the raw data, first by observing the peaks on the live chromatogram read out (see Fig. 1) and then by checking the values of the d4-SAH in the excel file output following peak integration, we have now implemented a Pipeline Pilot script that can be used to identify compound wells with an unusually high internal standard value (defined as any single well where the value obtained for d4-SAH exceeds 3 standard deviations over the median value measured for d4-SAH in that plate). Whilst this increases the speed with which these can be identified, allowing us to assess data from several 384 well plates in a few minutes instead of several minutes per plate, it also standardises the process rather than relying on subjective judgements which may vary from operator to operator.

## 4 Conclusion

In this work we have reported a previously unpublished potential mode of assay interference when using RapidFire MRM technology and offer suggested mitigating strategies to either identify or avoid this. The ideal internal standard is one that is chemically similar and with similar properties to the analyte being detected. This ensures that any fluctuations in the sampling or detection process would affect them both proportionately, allowing the correction of these variables by normalising the analyte to the standard. In this assay the enzymatic product of the methyltransferase, SAH is the analyte and the deuterated form, d4-SAH was selected as the internal standard. However, this analyte, and the corresponding standard, has a mass that is close to typical HTS screening compounds. There is a risk therefore that such a compound may produce a trace that is comparable with either the analyte or the standard. We have not so far identified any compounds that show a trace similar to the analyte, SAH. We have, however, found examples of such compounds which are not only similar in mass but also fragment in such a way as to be confounding in relation to the internal standard. Our data have shown how such compounds can in fact return a false positive dose response curve, which may remain unidentified until or unless the compounds are retested in an orthogonal assay format. In cases where the analyte and the internal standard are similar in molecular weight to the compounds being tested, either implementing an automated script to monitor the internal standard signal or flagging hit compounds with masses close to the internal standard is recommended to be included as an additional quality control layer. We have developed a pipeline pilot script which flags any wells where the internal standard exceeds 3 standard deviations over the median signal for the internal standard for that plate. In this way we are able to then go back to the raw data, confirm any apparent interference and the select the compound for further testing in the orthogonal assay. If a second internal standard with a molecular weight beyond the range of HTS compounds can be practically included in the experiment, that can help circumvent compound interference issues, whilst maintaining the utility of the chemically similar internal standard. Use of the automated script helps to retain the high-throughput nature of the RF-MRM by removing time consuming and subjective visual inspection of the data, meaning that a full day’s run of 384 well plates can be assessed in minutes, instead of minutes per plate. In conclusion, the RF-MRM screening method remains a powerful technique that is anticipated to be less susceptible than other methods to false-positives, however caution is advised when hit compounds are close in molecular weight to the internal standard.

## CRediT authorship contribution statement

**De Lin:** Writing – review & editing, Methodology, Investigation, Formal analysis, Conceptualization. **Lesley-Anne Pearson:** Writing – review & editing, Writing – original draft, Investigation, Formal analysis, Data curation. **Shamshad Ahmad:** Methodology, Investigation. **Sandra O’Neill:** Methodology, Investigation, Data curation. **John Post:** Methodology, Investigation. **Colin Robinson:** Investigation, Formal analysis. **Duncan E. Scott:** Writing – review & editing, Writing – original draft, Supervision. **Ian H. Gilbert:** Writing – review & editing, Supervision, Funding acquisition.

## Declaration of competing interest

The authors declare that they have no known competing financial interests or personal relationships that could have appeared to influence the work reported in this paper.

## Appendix Supplementary materials

Image, application 1
