# Supplementary material for: Overcoming a false-positive mechanism in RapidFire MRM-based high throughput screening
Source: SLAS Discov. 2025 Sep;35:None. doi: 10.1016/j.slasd.2025.100252 (PMC12823550; doi:10.1016/j.slasd.2025.100252)
Supplement: Supplementary file 1 [file mmc1.docx]

**Supporting information**

**Overcoming a false-positive mechanism in RapidFire MRM-based high throughput screening**

De Lin†, Lesley-Anne Pearson†, Shamshad Ahmad, Sandra O’Neill, John Post, Colin Robinson, Duncan E. Scott, Ian H. Gilbert*

† Joint first author * Corresponding author

Contents: Figures S1-S6


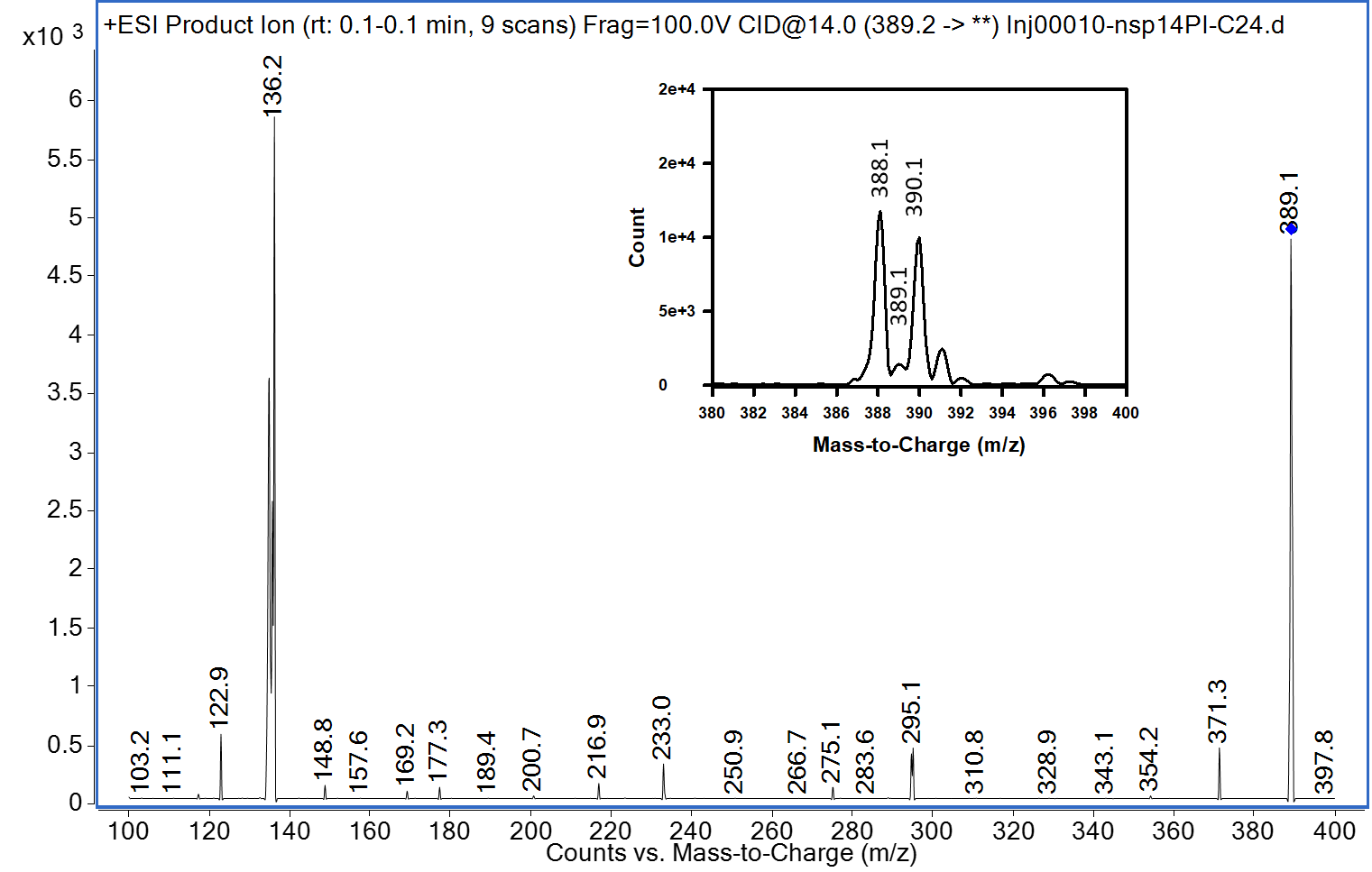


Figure S1: Compound 1 product ion scan of m/z 389.2, scans were acquired from m/z 100-400. The inset shows the full scan (m/z 380-400) with relative abundances of M (m/z 388.1), M+1 (m/z 389.1), and +2 (m/z 390.1) isotopes.


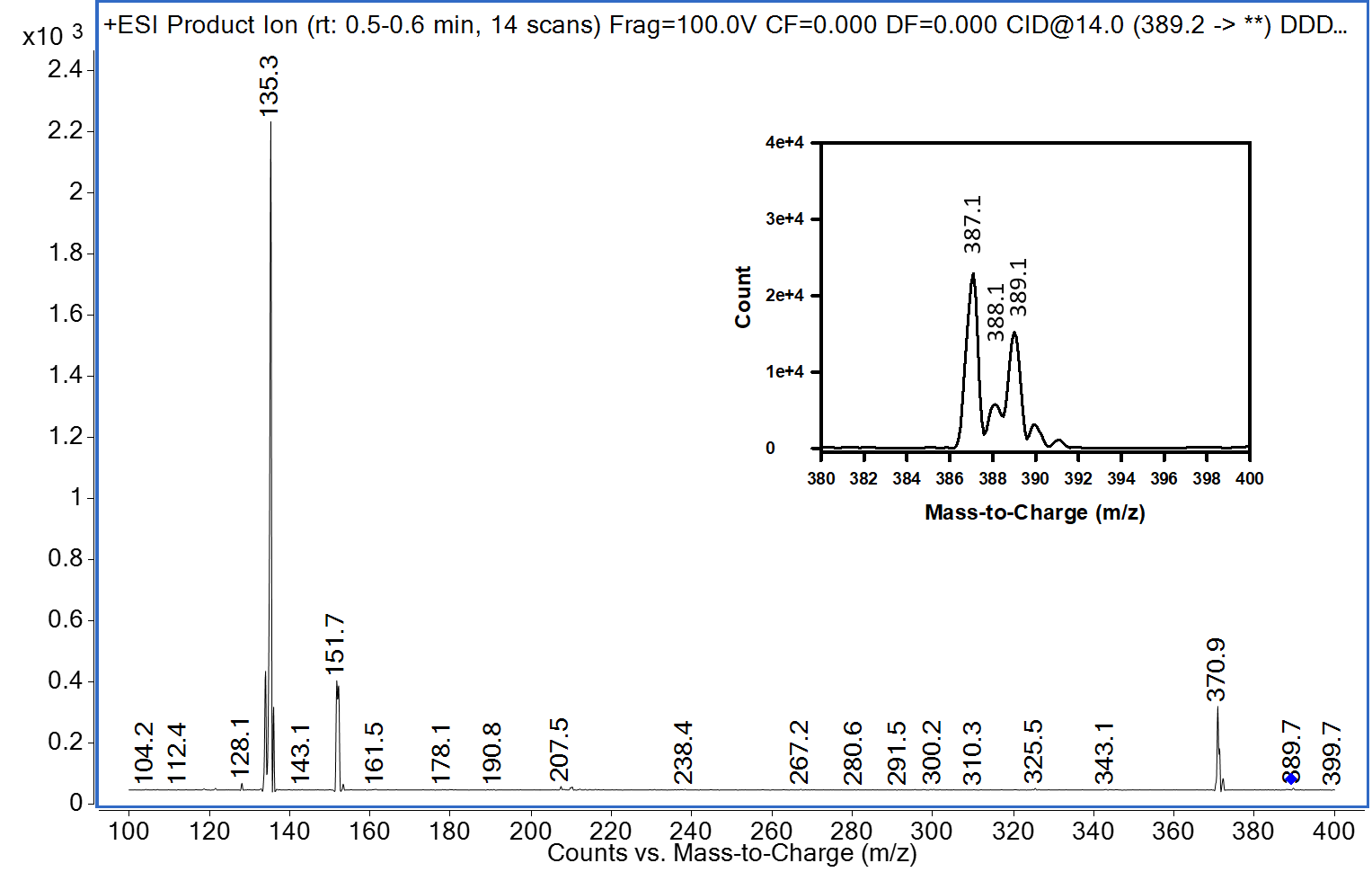


Figure S2: Compound 2 product ion scan of m/z 389.2, scans were acquired from m/z 100-400. The inset shows the full scan (m/z 380-400) with relative abundances of M (m/z 387.1), M+1 (m/z 388.1), and +2 (m/z 389.1) isotopes.


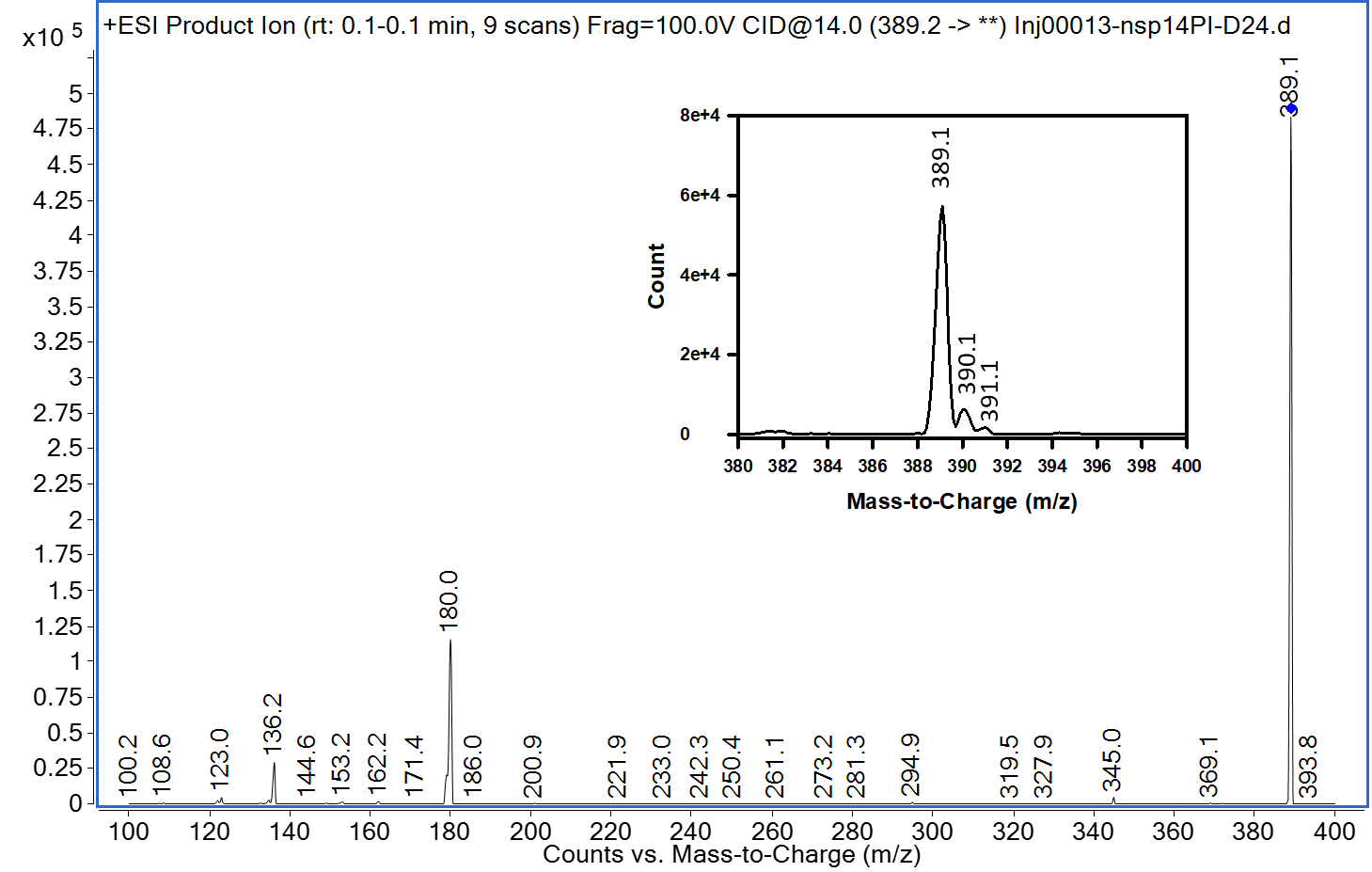


Figure S3: Compound 3 product ion scan of m/z 389.2, scans were acquired from m/z 100-400. The inset shows the full scan (m/z 380-400) with relative abundances of M (m/z 389.1), M+1 (m/z 390.1), and +2 (m/z 391.1) isotopes.


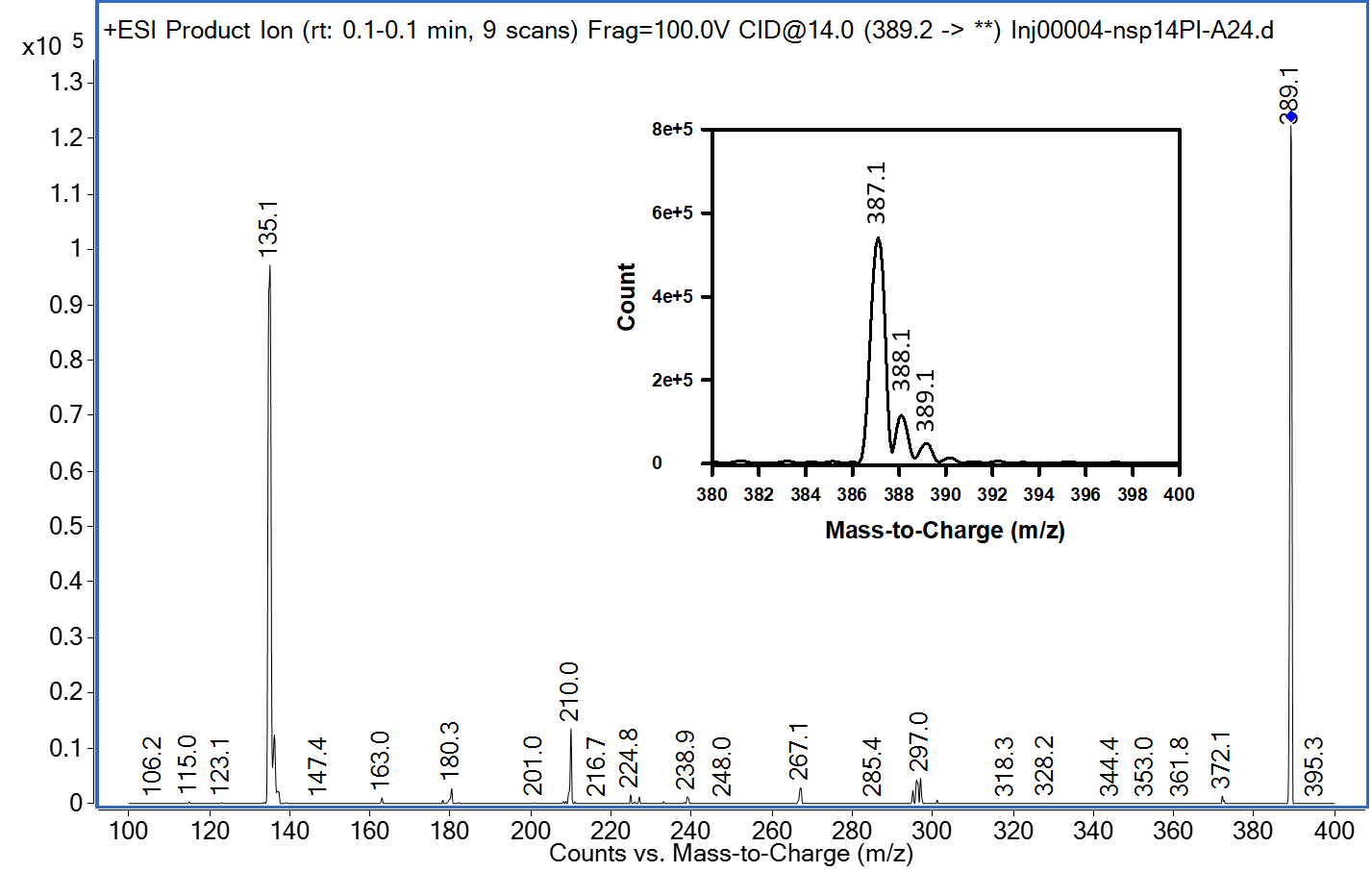


Figure S4: Compound 4 product ion scan of m/z 389.2, scans were acquired from m/z 100-400. The inset shows the full scan (m/z 380-400) with relative abundances of M (m/z 387.1), M+1 (m/z 388.1), and +2 (m/z 389.1) isotopes.


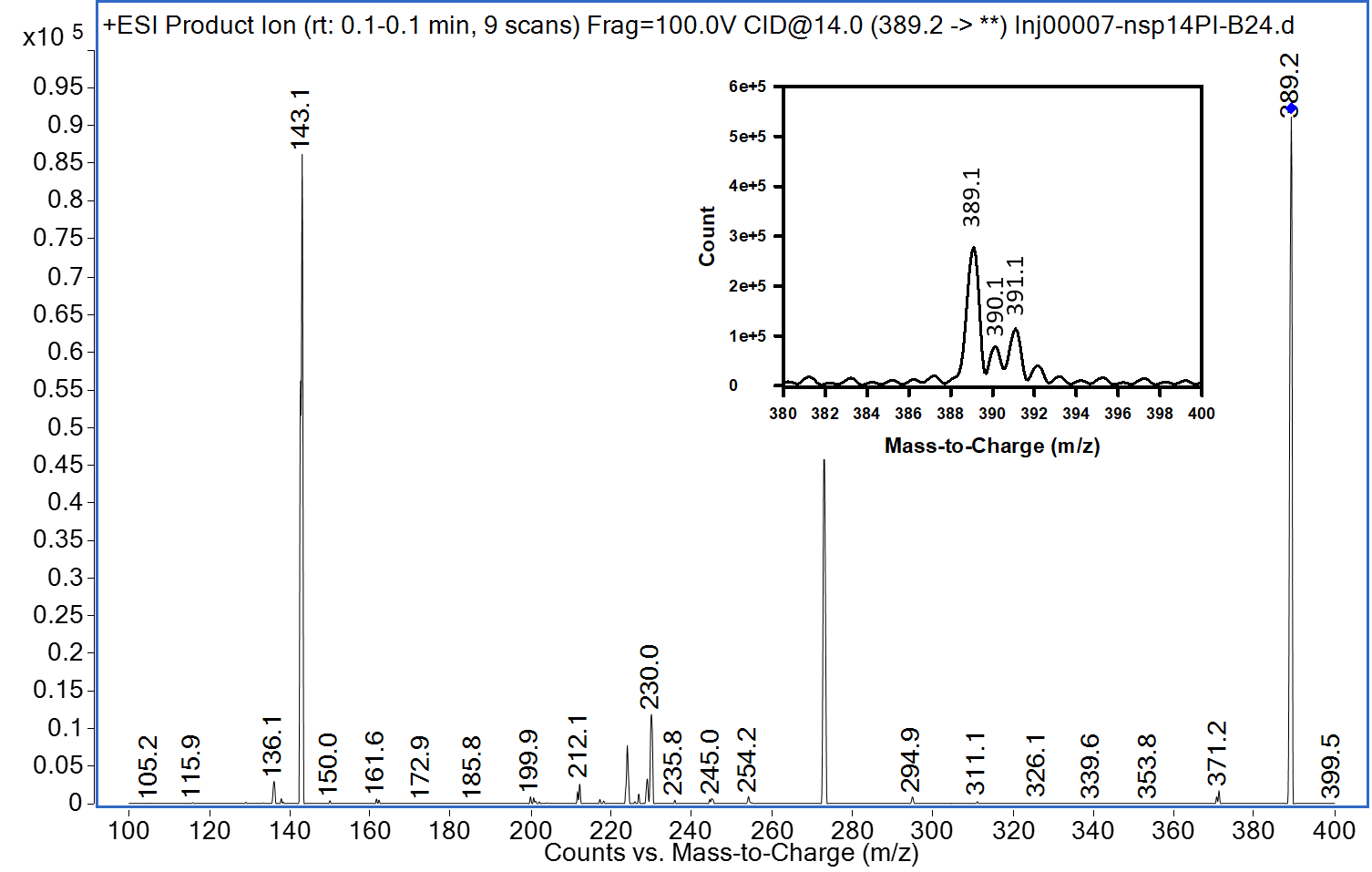


Figure S5: Compound 5 product ion scan of m/z 389.2, scans were acquired from m/z 100-400. The inset shows the full scan (m/z 380-400) with relative abundances of M (m/z 389.1), M+1 (m/z 390.1), and +2 (m/z 391.1) isotopes.


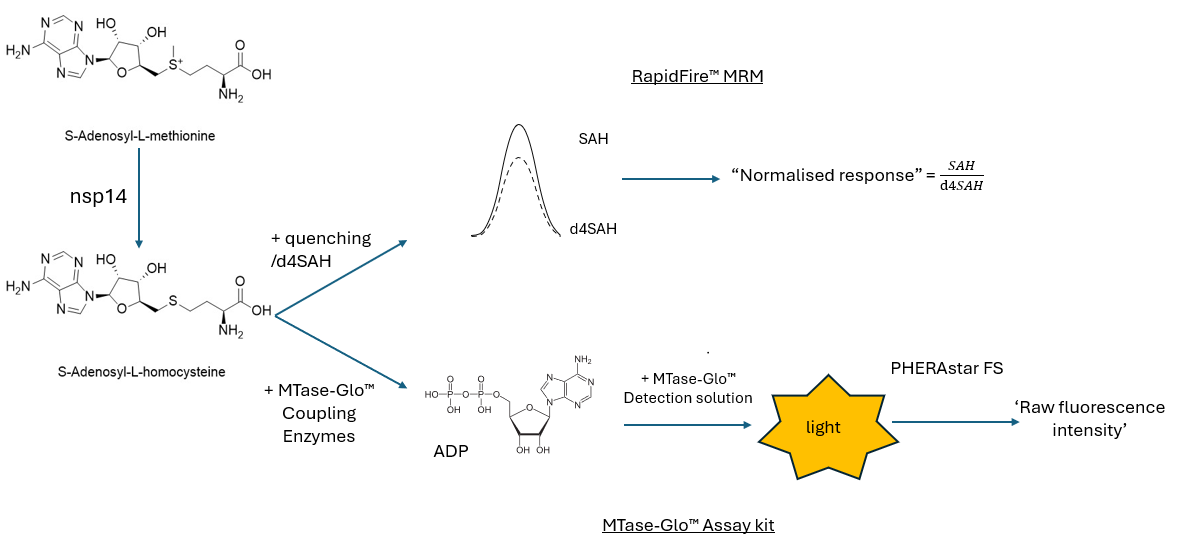


Figure S6: A workflow diagram comparing the assay processes for the RapidFire MRM and the MTase-Glo™ assay
